# Nutritional knowledge, dietary practice and associated factors among adults on antiretroviral therapy in Felege Hiwot referral hospital, Northwest Ethiopia

**DOI:** 10.1186/s40795-018-0256-5

**Published:** 2018-11-21

**Authors:** Getnet Mekuria Mengie, Tsegahun Worku, Amanuel Nana

**Affiliations:** Department of Applied Human Nutrition, Bahir Dar Institute of Technology, Bahir Dar University, Bahir Dar, Ethiopia

**Keywords:** HIV/AIDS, PLWHA, HAART, Nutritional knowledge, Dietary practice

## Abstract

**Background:**

Nutritional support is identified as one of the most critical and immediate needs for people living with human immunodeficiency virus/acquired immunodeficiency syndrome (HIV/AIDS). Adequate nutrition is vital to optimize response to medical treatment, manage opportunistic infections, maintain the immune system, and support optimal quality of life for people living with HIV/AIDS (PLWHA). Evidence has shown that the progression of the disease could be slowed with good nutrition. Nutrition interventions increase compliance with treatment regimens and optimize the benefits of antiretroviral drugs. The nutritional knowledge, dietary practice and associated factors among adults on antiretroviral therapy are not well understood generally in Ethiopia and particularly in Bahir Dar City. Therefore, the aim of this study was to assess the nutritional knowledge, dietary practice and associated factors among adult PLWHA on anti-retroviral therapy (ART) in Felege Hiwot Referral Hospital.

**Methods:**

Institution based cross-sectional study was conducted from April 13 to May 18, 2017 in Felege Hiwot Referral Hospital. Systematic random sampling technique was used to select 539 adults on highly active antiretroviral treatment (HAART). Data were collected using a semi-structured and pretested questionnaire. Bivariate and multivariate logistic regression analyses were carried out to identify factors associated with nutritional knowledge and dietary practice.

**Results:**

This study shows that 25.8, 52.5 and 21.7% of respondents had poor, average and good nutritional knowledge scores respectively. Ever heard about good nutrition and received dietary counselling were significant factors for nutritional knowledge. This study also reveals that 3.2, 66.4 and 30.4% of respondents had poor, average and good dietary practice scores respectively. Presence of gastrointestinal symptom, ever heard about good nutrition and good nutritional knowledge were significant factors for dietary practice.

**Conclusions:**

This study found that the magnitude of good nutritional knowledge and good dietary practice were 21.7 and 30.4%. Nutrition education and counseling should be given by health care workers for patients on ART to improve their nutritional knowledge. The media should also strengthen its role in disseminating nutrition information. The health professionals should routinely diagnose and treat gastrointestinal symptoms to maintain patients’ appetite for food their by increasing dietary intake.

## Background

Acquired immunodeficiency syndrome (AIDS) is a serious and fatal epidemic disease that comprises deficiency of the immune system, other complex and severe diseases leading to death throughout the world [1]. It is caused by a retrovirus, the human immunodeficiency virus (HIV), which attacks and impairs body’s natural defense system against disease and infection [2].

At the end of 2010, an estimated 34 million people were living with HIV throughout the world. In addition, a total of 2.7 million people acquired HIV infection during 2010, including 390,000 children less than 15 years. Worldwide, the annual number of people dying from AIDS related causes was 1.8 million in 2010 [3].

The HIV epidemic remains one of the major public health challenges especially for low and middle income countries. In sub-Saharan African countries, where most of the new HIV infections occurred, an estimated 1.9 million people became infected in 2010 [3]. According to Ethiopia Demographic and Health Survey (EDHS) 2011 preliminary report, 1.5% of adults age 15–49 were HIV infected in Ethiopia. The prevalence of HIV among women of reproductive age (15–49 years) was 1.9% and for men 15–49, it was 1% [4].

HIV/AIDS combined with malnutrition brought a significant crisis globally and in Sub-Saharan Africa particularly. HIV has an impact on households, communities and economic growth of nations. This virus specifically affects the health status of an individual. Malnutrition combined with infectious diseases aggravated the HIV/AIDS pandemic and contributed to morbidity and mortality of patients In most developing countries [5, 6].

In resource-limited settings, where malnutrition and food insecurity are endemic nutrition is an important component of comprehensive care for HIV-infected individuals. Malnutrition occurring before HIV infection i.e. pre-existing malnutrition compromises the immune system. The cellular effects of HIV on the immune system are similar to those of malnutrition. Theyinclude suppression of delayed hypersensitivity, decreases in CD4 T-cells and abnormal B-cell responses [7–10].

Nutritional support is identified as one of the most critical and immediate needs for people living with HIV/AIDS [11]. Adequate nutrition is vital to optimize response to medical treatment, manage opportunistic infections, maintain the immune system, and support optimal quality of life for people living with HIV/AIDS (PLWHA) [12]. Evidence has shown that the progression of the disease could be slowed with good nutrition. Nutrition interventions increase compliance with treatment regimens and optimize the benefits of antiretroviral drugs [13].

The nutritional knowledge, dietary practice and associated factors among adults on ART are not well understood generally in Ethiopia and particularly in Bahir Dar City. Therefore, this study is important to assess the nutritional knowledge, dietary practice and associated factors among adult PLWHA on ART in Felege Hiwot Referral Hospital, Northwest Ethiopia, 2017.

## Methods

### Study design, area and period

Institution based cross-sectional study was conducted from April 13, 2017 to May 18, 2017 in Felege Hiwot Referral Hospital which is located in Bahir Dar city. The hospital provides ART service starting from 1995 E.C. This hospital provides ART service for about 6144 people, and among these 6126 were on ART and 18 were on pre ART. There were 4 out patient units that provide ART service in the hospital with average visit of 80 patients daily. The total average visits to ART clinic monthly was 1500–1600 people. The source populations were all adult PLWHA on HAART registered and have a follow up for their treatment in Felege Hiwot Referral Hospital at the time of the study. The study populations were all adult PLWHA on HAART regardless of their treatment regimen and available during data collection period.

### Sample size and sampling technique

The sample size was calculated by using a single population proportion formula with the following considerations; prevalence of adequate nutritional knowledge (70.9%) from previous research conducted in Ghana [14], 95% confidence level, 4% margin of error and a 10% non-response rate. A total of 546 adult PLWHA on HAART were selected using systematic random sampling technique.

### Data collection procedures

Interviewer administered questionnaire was used to collect data from study subjects. First, the English version of the questionnaire was prepared by investigators. Then it was translated to Amharic version and back to English by language translators in order to check for consistency.

For assessment of nutritional knowledge and dietary practice, questions were adapted from a research done in Lagos Southwest Nigeria [15] and modified after pre-test. Reliability of the tool was checked using test-retest method. Questions with less than 0.7 kappa or Pearson coefficient values were removed or revised. The scoring system in each section was as follows:

Knowledge: ‘No=1, ‘don’t know’=0 and ‘Yes’=2. Scores were categorized as; 0–8 for ‘poor’, 9–14 for ‘average’ 15 and above for ‘good’. Nine questions were used to assess nutritional knowledge of respondents. The minimum attainable score is 0 and the maximum score is 18.

Practice: ‘No’ = 1, ‘undecided’ = 0 and ‘Yes’ = 2. Scores were categorized as; 0–5 for ‘poor’, 6–11 for ‘average’ 12 and above for ‘good’. Eight questions were used to assess dietary practice of respondents. The minimum attainable score is 0 and the maximum score is 16.

For data collection 10 diploma nurses and for supervision 2 BSc nurses were recruited and trained for one day before the data collection started. Supervisors closely monitored data collectors daily during data collection. In addition, investigators together with supervisors checked the collected data daily.

### Operational definitions

**Good nutritional knowledge**: if respondent nutrition knowledge score was 15 and above out of 18 nutritional knowledge assessment questions score.

**Good dietary practice**: if respondent dietary practice score was 12 and above out of 16 dietary practices assessment questions score.

**Ever heard about good nutrition**: if respondent heard information concerning balanced, safe, adequate and diversified food.

### Data processing and analysis

First, the collected data were checked for completeness then coded before entered in to EPI info. Next data from completed questionnaire was entered (double entry of 20% of the questionnaire) in to EPI INFO version 3.5.3 and transferred in to SPSS version 20 for analysis. Descriptive statistics were carried out in order to characterize respondents with different variables of interest. First, bivariate logistic regression analysis was done for each explanatory variable with outcome variables (nutritional knowledge and dietary practice). Then variables which had *P*-value ≤0.2 during the bivariate logistic regression analysis were entered in to multivariate logistic regression analysis. Statistical significance was determined using odds ratio with 95% confidence interval. Statistical significance was declared if *P*-value is < 0.05.

### Ethical consideration

Ethical clearance was obtained from Ethical Review Board of Bahir Dar University Institute of Technology. After this, supportive letter was written by Amhara Public Health Institute in order to conduct the research in the hospital. In addition, informed verbal consent was obtained from the respondents before interviewing. Respondents were told about the aim of the study and confidentiality of the information which they gave. In addition, they were told that they have full right to withdraw from the study at any time if they feel that uncomfortable. Confidentiality and privacy of collected information ensured at all levels.

## Results

### Socio-demographic characteristics

Out of 546 eligible study participants, 539 of them agreed to participate in this study, which made the response rate 98.7%. The mean age of the respondents was 37.2 years (standard deviation, SD ±8.74). Almost two third (62.2%) of respondents were in the age group of 30–44 years. More than half (52.3%) of them were females. With regard to religion, the majority (90.7%) of respondents were Orthodox Christian followers. Almost half (49.7%) of them were married. With regard to residence, the majority (93.1%) of respondents live in urban area. The remaining 6.9% of them live in rural area (Table 1).

**Table 1:** Socio-demographic characteristics of study participants: Bahir Dar Felege Hiwot Referral Hospital, Northwest Ethiopia April, 2018 (*n* = 539)

Variables	Frequency	Percentage
Sex		
Male	257	47.7
Female	282	52.3
Age		
18–29	89	16.5
30–44	335	62.2
> = 45	115	21.3
Religion		
Orthodox	489	90.7
Muslim	46	8.5
Protestant	4	0.7
Ethnicity		
Amhara	526	97.6
Awi	6	1.1
Others^a^	7	1.3
Education status		
Unable to read and write	35	6.5
Able to read and write	51	9.5
Primary	161	29.9
Secondary	139	25.8
Certificate/diploma	102	18.9
Degree and above	51	9.5
Occupation		
Housewife	102	18.9
Government employed	151	28.0
Private employed	68	12.6
Merchant	107	19.9
Daily laborer	87	16.1
Others^b^	24	4.5
Marital status		
Single	110	20.4
Married	268	49.7
Widowed	72	13.4
Divorced	85	15.8
Separated	4	0.7
Residence		
Urban	502	93.1
Rural	37	6.9
Monthly income		
<=500 birr	47	8.7
501–1000 birr	152	28.2
> 1000 birr	340	63.1

^a^Tigre, Oromo

^b^Farmer, student, housemaid, driver, mechanic, pensioner, jobless, carpenter, masonry

### Clinical and immunological characteristics

Nearly one fifth (19.7%) of respondents had eating problems. The commonest eating problems were vomiting (50.9%), loss of appetite (16.1%), both vomiting and loss of appetite (29.2%). More than one fourth (25.4%) of respondents had gastrointestinal symptoms. The commonest gastrointestinal symptoms were diarrhea (37.2%), indigestion (35.0%) and constipation (22.7%). Almost half (47.9%) of respondents encountered opportunistic infections. The most common opportunistic infections were TB (63.6%), oral lesion (15.5%) and chronic diarrhea (12.0%).

With regard to WHO stage of HIV; 38.4, 15.6, 39.7 and 6.3% of respondents were stage I, II, III and IV respectively (Table 2).

**Table 2:** Clinical and immunological characteristics of study participants: Bahir Dar Felege Hiwot Referral Hospital, Northwest Ethiopia April, 2018 (n = 539)

Variables	Frequency	Percentage
Presence of eating problem		
Yes	106	19.7
No	433	80.3
Type of eating problem (*n* = 106)		
Loss of appetite	17	16.1
Vomiting	54	50.9
Loss of appetite plus vomiting	31	29.2
Others^a^	4	3.8
Presence of GI symptoms		
Yes	137	25.4
No	402	74.6
Type of GI symptoms (*n* = 137)		
Diarrhea	51	37.2
Indigestion	48	35.0
Constipation	31	22.7
Others^b^	7	5.1
Presence of opportunistic infection		
Yes	258	47.9
No	281	52.1
Type of opportunistic infection (*n* = 258)		
TB	164	63.6
Oral lesion	40	15.5
Chronic diarrhea	31	12.0
Others^c^	23	8.9
WHO stage of HIV		
Stage I	207	38.4
Stage II	84	15.6
Stage III	214	39.7
Stage IV	34	6.3
CD4 count		
< 200 cells	44	8.2
200–350 cells	164	30.4
> 350 cells	331	61.4
Patients current treatment regimen		
1c (AZT + 3TC + NVP)	145	26.9
1d (AZT + 3TC + EFV)	65	12.1
1e (TDF + 3TC + EFV)	191	35.4
1f (TDF + 3TC + NVP)	43	8.0
2 h (TDF + 3TC + Atazanavir)	55	10.2
2f (AZT + 3TC + Atazanavir)	22	4.1
Others ^d^	18	3.3

^a^=Swallowing difficulty, chest pain

^b^=Stomach pain, bloating, flatulence

^c^=Toxoplasmosis, herpes zoster, intestinal TB

^d^=AZT + 3TC + ABC, ABC + 3TC + EFV, ABC + 3TC + Atazanavir

### Behavioral and nutrition related characteristics

Almost all (98.0%) of respondents didn’t smoke cigarettes. More than half (53.8%) of them didn’t heard about good nutrition. Two hundred thirty one (42.9%) of them received dietary counseling. Only 9.1% of them received nutritional support (Table 3).

**Table 3:** Behavioural and nutrition related characteristics of study participants: Bahir Dar Felege Hiwot Referral Hospital, Northwest Ethiopia April, 2018. (*n* = 539)

Variables	Frequency	Percentage
Cigarette smoking		
Yes	11	2.0
No	528	98.0
Ever heard about good nutrition		
Yes	249	46.2
No	290	53.8
Received dietary counselling		
Yes	231	42.9
No	308	57.1
Received nutritional support		
Yes	49	9.1
No	490	90.9

### Nutritional knowledge of study participants

The overall nutritional knowledge score (mean ± SD) of the respondents was 11.2 ± 3.7, which lied in the average category. The results of this study showed that more than half (52.5%) of them had average nutritional knowledge. But only 21.7% of them had good knowledge about nutrition. Three hundred eighty three (71.1%) of respondents knew that carbohydrates and fats are energy giving nutrients. Three hundred twenty three (59.9%) of them didn’t know that frequent stooling leads to nutrient loss (Table 4).

**Table 4:** Nutritional knowledge of study participants: Bahir Dar Felege Hiwot Referral Hospital, Northwest Ethiopia April, 2018. (n = 539)

Statement	Yes n (%)	No n (%)	Don’t know n (%)
Carbohydrates and fats are energy-giving foods	383(71.1)	53(9.8)	103(19.1)
Balanced diet is important to prevent infection	416(77.2)	71(13.2)	52(9.6)
Fish is a good source of protein	417(77.4)	58(10.8)	64(11.9)
Frequent stooling leads to nutrient loss	94(17.4)	122(22.6)	323(59.9)
Protein- rich foods are needed to build and repair body tissues	397(73.7)	95(17.6)	47(8.7)
HIV infection increases body’s food requirement	256(47.5)	164(30.4)	119(22.1)
Water is a nutrient	153(28.4)	150(27.8)	236(43.8)
Banana is good for control of diarrhea in HIV patients	116(21.5)	241(44.7)	182(33.8)
Fruits and vegetables are rich in vitamins and minerals	250(46.4)	121(22.4)	168(31.2)
Over all nutritional knowledge score			
Poor	139(25.8)		
Average	283(52.5)		
Good	117(21.7)		

### Dietary practice of study participants

The overall dietary practice score (mean ± SD) of the respondents was 10.4 ± 2.7, which lied in the average category. The findings of this study revealed that two third (66.4%) of respondents had average dietary practice. However, lower than one third (30.4%) of them had good dietary practice. More than half (53.6%) of respondents didn’t eat balanced meals regularly. Almost half (49.5%) of them consumed variety of foods in moderation (Table 5).

**Table 5:** Dietary practice of study participants: Bahir Dar Felege Hiwot Referral Hospital, Northwest Ethiopia April, 2018. (*n* = 539)

Question	Yes n (%)	No n (%)	Undecided n (%)
Do you usually eat washed raw vegetables more than cooked vegetables?	149(27.6)	338(62.7)	52(9.6)
Do you know your nutritional needs differ from non-HIV positive persons so you made an effort to eat a balanced diet?	300(55.7)	195(36.2)	44(8.2)
Do you usually eat fruits?	160(29.7)	273(50.6)	106(19.7)
Do you eat balanced meals regularly?	127(23.6)	289(53.6)	123(22.8)
Do you maintain good nutrition despite your income?	147(27.3)	250(46.4)	142(26.3)
Do you take herbs along with your drugs?	38(7.1)	483(89.6)	18(3.3)
Do you eat a variety of foods in moderation?	267(49.5)	220(40.8)	52(9.6)
Do you engage in good eating habits in order to maintain your overall health?	247(45.8)	214(39.7)	78(14.5)
Over all dietary practice score			
Poor	17(3.2)		
Average	358(66.4)		
Good	164(30.4)		

### Factors associated with nutritional knowledge

Hosmer-Lemeshow goodness-of-fit test (*p* = 0.311) was used to assess the fitness of the model. In the bivariate logistic regression model; place of residence, ever heard about good nutrition and received dietary counselling were significantly associated with nutritional knowledge.

In the multivariate logistic regression model; ever heard about good nutrition and received dietary counselling were significantly associated with nutritional knowledge. But, place of residence was not significantly associated with nutritional knowledge (Table 6). The odds of having good nutritional knowledge is 1.94 times higher among patients who ever heard about good nutrition compared to those didn’t ever hear [AOR = 1.94(1.003,3.761)]. The odds of having good nutritional knowledge is 6.15 times higher among patients who received dietary counseling compared to those who didn’t receive [AOR = 6.15(3.134, 12.063)].

**Table 6:** Bivariate and multivariate logistic regression analysis showing factors associated with nutritional knowledge of adults on ART in Bahir Dar Felege Hiwot Referral Hospital, Northwest Ethiopia April, 2018

Variables	Good nutritional Knowledge Yes No		COR 95% CI	AOR 95% CI	P-value
Residence					
Urban	113(21.0)	389(72.2)	2.39(0.83,6.91)		
Rural	4(0.7)	33(6.1)	1.00		
Ever heard about good nutrition					
Yes	93(17.3)	156(28.9)	6.61(4.05,10.79)	1.94(1.003,3.761)	0.049
No	24(4.5)	266(49.4)	1.00	1.00	
Received dietary counselling					
Yes	96(17.8)	135(25.0)	9.72(5.81,16.26)	6.15(3.134,12.063)	0.000
No	21(3.9)	287(53.2)	1.00	1.00	

*COR*: Crude Odds Ratio

### Factors associated with dietary practice

Hosmer-Lemeshow goodness-of-fit test (*p* = 0.822) was used to assess the fitness of the model. In the bivariate logistic regression model; presence of eating problem, presence of gastrointestinal symptoms, presence of opportunistic infections, ever heard about good nutrition, received dietary counselling, received nutritional support and good nutritional knowledge were significantly associated with dietary practice.

In the multivariate logistic regression model; presence of gastrointestinal symptoms, ever heard about good nutrition and good nutritional knowledge were significantly associated with dietary practice. But, presence of eating problem, presence of opportunistic infections, received dietary counselling and received nutritional support were not significantly associated with dietary practice (Table 7). The odds of having good dietary practice is 2.28 times higher among patients who hadn’t gastrointestinal symptom compared to those who had gastrointestinal symptom [AOR = 2.28(1.39, 3.78)]. The odds of having good dietary practice is 2.14 times higher among patients who ever heard about good nutrition compared to those didn’t ever hear [AOR = 2.14(1.41,3.27)]. The odds of having good dietary practice is 3.74 times higher among patients who had good nutritional knowledge compared those who hadn’t good nutritional knowledge [AOR = 3.74(2.33, 6.01)].

**Table 7:** Bivariate and multivariate logistic regression analysis showing factors associated with dietary practice of adults on ART in Bahir Dar Felege Hiwot Referral Hospital, Northwest Ethiopia April, 2018

Variables	Good dietary practice Yes No		COR 95% CI	AOR 95% CI	P-value
Presence of eating problem					
Yes	22(4.1)	84(15.6)	1.86(1.12,3.10)		
No	142(26.3)	291(54.0)	1.00		
Presence of GI symptoms					
Yes	28(5.2)	109(20.2)	1.00	1.00	
No	136(25.2)	266(49.4)	1.99(1.25,3.17)	2.28(1.39,3.78)	0.001
Presence of opportunistic infection					
Yes	69(12.8)	189(35.1)	1.00		
No	95(17.6)	186(34.5)	1.39(0.97,2.03)		
Ever heard about good nutrition					
Yes	105(19.5)	144(26.7)	2.86(1.95,4.18)	2.14(1.41,3.27)	0.000
No	59(10.9)	231(42.9)	1.00	1.00	
Received dietary counselling					
Yes	98(18.2)	133(24.7)	2.70(1.85,3.94		
No	66(12.2)	242(44.9)	1.00		
Received nutritional support					
Yes	19(3.5)	30(5.6)	1.51(0.82,2.76)		
No	145(26.9)	345(64.0)	1.00		
Good nutritional knowledge					
Yes	67(12.4)	50(9.3)	4.49(2.92,6.91)	3.74(2.33,6.01)	0.00
No	97(18.0)	325(60.3)	1.00	1.00	

*COR*: Crude Odds Ratio

## Discussion

This institution based cross sectional study assessed the nutritional knowledge, dietary practice and associated factors among adult PLWHA on ART in Felege Hiwot Referral Hospital, Northwest Ethiopia, 2017. In resource-limited settings, nutrition is an important component of comprehensive care for HIV-infected individuals.

The results of this study showed that poor, average and good nutritional knowledge scores of adults on ART in Felege Hiwot Referral Hospital were found to be 25.8, 52.5 and 21.7% respectively. Previous study (Nti CA et al., 2012) conducted in Ghana reported that 9.1, 20.0 and 70.9% of the repondents had poor, fair and adequate nutritional knowledge scores respectively [14]. Ezechi L, Brai B, Osifeso G, Mbah P and Ezechi O [15] reported that 11.7, 64.8 and 23.5% of women living with HIV/AIDS in Lagos, Southwest Nigeria had poor, average and good nutritional knowledge scores. Anand D and Puri S [16] revealed that 12.0, 71.2 and 16.8% of PLHIV from New Delhi, India had poor, average and good nutritional knowledge scores. This difference might be due to differences in socioecomic status, health seeking behaviour and health care system.

Ever heard about good nutrition is among the nutrition related factors that shows significant association with nutritional knowledge in this study. The odds of having good nutritional knowledge is 1.94 times higher among patients who ever heard about good nutrition compared to those didn’t ever hear. This could be explained by the fact that exposure to information concerning good nutrition ultimately improves nutritional knowledge of human beings.

Receiving dietary counselling is another nutrition related factor that has a significant association with nutritional knowledge. The odds of having good nutritional knowledge is 6.15 times higher among patients who received dietary counseling compared to those who didn’t receive. This could be due to the fact that dietary counselling is one of the strategies to increase nutritional knowledge of individuals.

This study also found that poor, average and good dietary practice scores of adults on ART were found to be 3.2, 66.4 and 30.4% respectively. Previous study (Nti CA et al., 2012) conducted in Ghana reported that 50.0, 41.8 and 8.2% of the repondents had poor, fair and adequate dietary practice scores respectively [14]. Ezechi L, Brai B, Osifeso G, Mbah P and Ezechi O [15] reported that 1.9, 33.0 and 65.1% of women living with HIV/AIDS in Lagos, Southwest Nigeria had poor, average and good dietary practice scores. This difference might be due to differences in culture, time, belief, attitude, life style, socioecomic status and knowledge about food as well as nutrition.

Presence of gastrointestinal symptom is among various clinical factors that shows significant association with dietary practice in this study. The odds of having good dietary practice is 2.28 times higher among patients who hadn’t gastrointestinal symptom compared to those who had gastrointestinal symptom.This could be due to the occurrence of one or more gastrointestinal symptoms may contribute to loss appetite which decreases dietary intake.

Ever heard about good nutrition is among the nutrition related factors that has a significant association with dietary practice. The odds of having good dietary practice is 2.14 times higher among patients who ever heard about good nutrition compared to those didn’t ever hear. This could be explained by the fact that exposure to information concerning good nutrition increases nutritional knowledge that ultimately improves dietary intake of individuals.

Good nutritional knowledge is significantly associated with dietary practice in this study. The odds of having good dietary practice is 3.74 times higher among patients who had good nutritional knowledge compared those who hadn’t good nutritional knowledge.This finding is in line with study conducted by Ezechi L, et al., 2016 [15] in Lagos, Southwest Nigeria. This could be due to the fact that good nutritional knowledge is one of the prerequisites for ensuring good dietary practice.

The strength of this study is two out comes i.e. nutritional knowledge and dietary practice are assessed. The other strength is multiple associated factors are assessed. The limitations of this study are social desirability and recall bias when measuring nutritional knowledge and dietary practice.

## Conclusion and recommendations

The findings of this study shows that 25.8, 52.5 and 21.7% of respondents had poor, average and good nutritional knowledge scores respectively. Ever heard about good nutrition and received dietary counselling were significant factors for nutritional knowledge. Nutrition education and counseling should be given by health care workers for patients on ART to improve their nutritional knowledge. The media should also strengthen its role in disseminating nutrition information.

This study also reveals that 3.2, 66.4 and 30.4% of respondents had poor, average and good dietary practice scores respectively. Presence of gastrointestinal symptom, ever heard about good nutrition and good nutritional knowledge were significant factors for dietary practice. The health professionals should routinely diagnose and treat gastrointestinal symptoms to maintain patients’ appetite for food their by increasing dietary intake. In addition, improving nutritional knowledge by providing nutrition education as well as nutrition information dissemination via various media outlets to sustain and increase dietary practice of patients.

## Abbreviations

AIDS: Acquired Immunodeficiency Syndrome
AOR: Adjusted Odds Ratio
ART: Antiretroviral Treatment
CD4: Cluster of Differentiation
CI: Confidence Interval
COR: Crude Odds Ratio
E.C: Ethiopian Calendar
EDHS: Ethiopian Demographic and Health Survey
HAART: Highly Active Antiretroviral Treatment
HIV: Human Immunodeficiency Virus
PLWHA: People Living With HIV/AIDS
SD: Standard Deviation
SPSS: Statistical Package for Social Sciences
